# Editorial: Managing healthcare transformation towards P5 medicine

**DOI:** 10.3389/fmed.2023.1244100

**Published:** 2023-08-25

**Authors:** Bernd Blobel, Dipak Kalra

**Affiliations:** ^1^Medical Faculty, University of Regensburg, Regensburg, Germany; ^2^eHealth Competence Center Bavaria, Deggendorf Institute of Technology, Deggendorf, Germany; ^3^First Medical Faculty, Charles University Prague, Prague, Czechia; ^4^The European Institute for Innovation Through Health Data, Ghent, Belgium

**Keywords:** personalized medicine, healthcare transformation, health ecosystem, precision medicine, translational medicine, P5 medicine

After publishing a paper on “Challenges and solutions for designing and managing pHealth ecosystems” (1) in the Frontiers in Medicine Research Topic “Personal Health Systems”, Frontiers in Medicine invited Bernd Blobel to edit a related Research Topic on the paper's topic. Contrary to traditional Frontiers in Medicine Research Topics based on open calls, the intended highly interdisciplinary volume is designed as a foundational textbook, established by papers on invitation only, that way guaranteeing a comprehensive, consistent and interrelated set of chapters. Therefore, Blobel et al. as Editor of the Research Topic “Managing healthcare transformation towards 5P medicine” first framed the topic regarding the objectives and challenges of transformed health ecosystems, their structures and functions, the involved domains including their methodologies and knowledge representation styles, as well as enabling technologies in a multidisciplinary approach to the transformation of health and social systems. After having defined the titles of the chapters to be included, he approached the internationally most acknowledged experts on those defined topics. Due to the special nature of the volume, a specific editor and reviewer pool had to be established first. Thus, he appointed Dipak Kalra as Research Topic Co-Editor. All papers have been first submitted to the Editor for check, harmonization, completion, etc., before running the formal submission process, followed by the formal review managed by the Co-Editor. Following the ethical rules of Frontiers, for all papers with the involvement of one of the editors as co-author, George Mihalas has been appointed as Research Topic Guest Editor. Without the latter's incredible engagement, the volume at hand wouldn't have been realizable.

Health and social care systems around the world are facing radical organizational, methodological and technological paradigm changes to meet the requirements for responding cost-effectively to increasing health demands, improving quality and safety of care, efficiency and efficacy of care processes and strengthening health systems resilience post-COVID. In this context, they are trying to tackle—usually without increased budgets—the challenges of ongoing demographic changes toward aging, multi-diseased societies, development of human resources, a health and social services consumerism, medical and biomedical progress, and exploding costs for health-related R&D as well as health services delivery. Furthermore, they intend to achieve sustainability of global health systems by transforming them toward intelligent, adaptive and proactive systems focusing on health and wellness with optimized quality and safety outcomes.

The targeted outcome is a transformed health and wellness ecosystem combining the approaches of translational medicine, 5P medicine (personalized, preventive, predictive, participative precision medicine) and digital health toward ubiquitous personalized health services realized independent of time and location, preferably more strongly engaging and empowering the patient and citizen in maintaining their own health. It considers individual health status, conditions, genetic and genomic dispositions in personal social, occupational, environmental and behavioral context, thus turning health and social care from reactive to proactive. This requires the advancement communication and cooperation among the business actors from different domains (disciplines) with different methodologies, terminologies/ontologies, education, skills and experiences from data level (data sharing) to concept/knowledge level (knowledge sharing). The challenge here is the understanding and the formal as well as consistent representation of the world of sciences and practices, i.e., of multidisciplinary and dynamic systems in variable context, for enabling mapping between the different disciplines, methodologies, perspectives, intentions, languages, etc. This co-operation amongst disciplines and perspectives is vital if we are to correctly and successfully develop and deploy increasingly sophisticated digital solutions in increasingly complex health and care systems. Based on a framework for dynamically, use-case-specifically and context-aware representing multi-domain ecosystems including their development process, systems, models and artifacts can be consistently represented, harmonized and integrated.

The response to that problem is the formal representation of health and social care ecosystems through a system-oriented, architecture-centric, ontology-based and policy-driven model and framework, addressing all domains and development process views contributing to the system and context in question (Blobel et al.). The representational challenges regarding ontologies and linguistics are specifically addressed (Kreuzthaler et al.). Such transformed health ecosystems must be designed and implemented in a secure and trustworthy way (Ruotsalainen et al.), meeting ethical requirements and principles (Maeckelberghe et al.). For providing implementable solutions and realizing them, the system must be properly modeled (Oemig and Blobel).

The described methodological paradigm changes must be accompanied by technological ones to enable healthcare transformation toward intelligent and increasingly autonomous ecosystems. Here, artificial intelligence (AI) and robotics must be mentioned (Denecke et al.). A special challenge of P5 medicine is the deployment of digital therapeutics. Their adoption and related success factors are specifically considered by Prodan et al..

This Frontiers Research Topic concludes with practical demonstrators such as healthcare transformation in low- and middle-income countries by using artificial intelligence (López et al.) or the deployment of the described methodologies in EU projects with a focus on SARS-CoV-2 pandemic (Paleari et al.).

## Author contributions

BB drafted the editorial. Both authors made substantial contributions to the work and approved it for publication.
